# Scaling up ART adherence clubs in the public sector health system in the Western Cape, South Africa: a study of the institutionalisation of a pilot innovation

**DOI:** 10.1186/s12992-018-0351-z

**Published:** 2018-04-25

**Authors:** Hayley MacGregor, Andrew McKenzie, Tanya Jacobs, Angelica Ullauri

**Affiliations:** 1 Institute of Development Studies, University of Sussex, Brighton, England; 2 DAI (formerly Health Partners International), Cape Town, South Africa; 3 Cape Town, South Africa; 4 Faculty of Health Sciences, University of Cape Town, Cape Town, South Africa

**Keywords:** HIV, South Africa, Health system, Complex adaptive systems, Innovation, Scaling up, Chronic illness, Differentiated HIV care, ART adherence

## Abstract

**Background:**

In 2011, a decision was made to scale up a pilot innovation involving ‘adherence clubs’ as a form of differentiated care for HIV positive people in the public sector antiretroviral therapy programme in the Western Cape Province of South Africa. In 2016 we were involved in the qualitative aspect of an evaluation of the adherence club model, the overall objective of which was to assess the health outcomes for patients accessing clubs through epidemiological analysis, and to conduct a health systems analysis to evaluate how the model of care performed at scale. In this paper we adopt a complex adaptive systems lens to analyse planned organisational change through intervention in a state health system. We explore the challenges associated with taking to scale a pilot that began as a relatively simple innovation by a non-governmental organisation.

**Results:**

Our analysis reveals how a programme initially representing a simple, unitary system in terms of management and clinical governance had evolved into a complex, differentiated care system. An innovation that was assessed as an excellent idea and received political backing, worked well whilst supported on a small scale. However, as scaling up progressed, challenges have emerged at the same time as support has waned. We identified a ‘tipping point’ at which the system was more likely to fail, as vulnerabilities magnified and the capacity for adaptation was exceeded. Yet the study also revealed the impressive capacity that a health system can have for catalysing novel approaches.

**Conclusions:**

We argue that innovation in largescale, complex programmes in health systems is a continuous process that requires ongoing support and attention to new innovation as challenges emerge. Rapid scaling up is also likely to require recourse to further resources, and a culture of iterative learning to address emerging challenges and mitigate complex system errors. These are necessary steps to the future success of adherence clubs as a cornerstone of differentiated care. Further research is needed to assess the equity and quality outcomes of a differentiated care model and to ensure the inclusive distribution of the benefits to all categories of people living with HIV.

## Background

### An innovation: The ART adherence club model

The South African public sector antiretroviral therapy (ART) programme, for people infected by Human Immunodeficiency Virus (HIV), has received earmarked funds and dedicated staffing. After a notoriously slow initiation of a government response to the HIV epidemic, the expansion of the ART programme has more recently been feted as a remarkable success story. A recent study found that all-cause mortality rates of patients on ART are similar to comparable cohorts in North America, despite the much higher levels of poverty and social disadvantage in South Africa [1]. The programme has also garnered attention for significant organisational innovation, such as a health management information system (HMIS), decentralised drug distribution, a routine clinical audit mechanism, community-based follow-up, and cohort registers and protocol-driven clinical records [2–5].

In this paper, we reflect upon the process whereby a pilot innovation, stemming from pioneering initiatives to develop a model of ‘differentiated care’ for HIV, became instituted in government policy. The innovation has been implemented within the public sector ART programme in HIV clinics, and scaled up and institutionalised within the respective state health facilities, spreading across an entire metropolitan area. Our interest is in analysing planned organisational change through intervention in a state health system, and in exploring the challenges associated with taking to scale a pilot that began as a relatively simple innovation by a non-governmental organisation (NGO). How do different stakeholders initially frame the need for innovation and what are the implications? What is the broader political, social and health systems context in which an innovation becomes policy, and what factors might enable or inhibit the change required for scaling up? These questions are posed for a scenario where change was introduced in the context of an existing large-scale national ART programme, a vertical service that by 2016 was assessed by many to be remarkable and to have successfully scaled up medication delivery in the country to millions of people, against considerable odds [6].

In early 2016, the national ART programme was considered the largest in the world and there were already an estimated 3.4 million HIV positive people on treatment in the public sector across South Africa [7]. This number was set to rise following a decision to adopt the World Health Organisation’s (WHO) ‘test and treat’ guidelines from late 2016. The sheer size of the programme creates challenges, and a further concern has been to keep people engaged in healthcare and adherent to medication. ‘Retention-in-care’, a measure of overall adherence, has declined as the programme has ‘matured’^1^ [3]. A study in urban Cape Town of hospital admissions for HIV, showed that a significant percentage of people had interrupted therapy. For those on ART at the time, nearly 50% were not virologically suppressed [8]. These remain the problems exercising HIV policy-makers and catalysing rhetoric for seeking ‘innovations’ in healthcare.

The Western Cape Province is seen to be better resourced than other provinces and the Department of Health considers itself a leader nationally in pioneering innovations, often working in conjunction with civil society organisations in piloting new ways of programme organisation. For instance, the international NGO Médecins Sans Frontières (MSF) has been involved in ART provision in low-income urban areas since before the state’s roll out of universal access in 2004. MSF thus has a longstanding reputation for spearheading initiatives aimed at improving ART provision and community-based care in high burden, low-resource settings (see for example [9, 10]).

As treatment expanded in the public sector in the mid-2000s, attention was drawn to the large numbers of HIV positive people (sometimes thousands) in high burden facilities, cycling regularly through the HIV clinics for routine care. Key stakeholders started to question whether HIV care could be further decentralised, and whether the same level of care was necessary for all. Various initiatives were tried by government staff and by NGOs to institute new ways of working, based on the idea of ‘differentiated care’. Here the key assumption is that some people can be identified as more stable from a clinical point-of-view, and triaged for less frequent monitoring and specialist clinical oversight [11–14].

In 2007, MSF started a pilot intervention devised to institute differentiated care for HIV positive adults on ART in a high-burden facility in one district of the metropolitan area of Cape Town. ART ‘adherence clubs’ were introduced within the ART programme. The adherence club (hereafter ‘club’) as a model involves identifying a group of people on ART as ‘stable’ and eligible for entry into a ‘club’; decentralising their care, including extending to spaces outside the main clinic; task shifting to community health workers (CHWs); streamlining of medication dispensing; and reducing the number of physician and blood-taking appointments. In 2011, clubs were adopted into policy by the Western Cape Provincial Department of Health (WCDoH). An intervention to extend them across the Cape Town metropole was implemented jointly with the City of Cape Town health department (CCTDoH).^2^

In 2016, we were involved in a mixed method evaluation of these clubs in the ART programme in Cape Town. We report here on our findings from the qualitative, health systems component of this evaluation, which was conducted within a sub-set of the facilities enrolled in a parallel epidemiological component. At the time of our study, the club model was already considered a success, and was thought to provide a way of service provision that was more convenient for ‘users’, and less burdensome for services. There was also talk of expansion beyond the Western Cape to other provinces. The model had attracted attention from the Bill and Melinda Gates Foundation on account of its perceived relevance for other high burden, low-resource settings. Funding for the evaluation arose from this interest. A health systems study was considered a necessary addendum to an epidemiological evaluation in order to understand better the process, and the successes and challenges, of scaling up the clubs. It was also considered important to distil and document the essence of the model in terms of its health system components, in order to enable the diffusion of the innovation to other geographic areas, and across to other chronic disease programmes. The club innovation is a pertinent example of a partnership between a NGO and a government, where it became possible to highlight an initiative as innovative, and argue for its institutionalisation. It was hoped that our health systems analysis would also address questions about the longer-term sustainability of a model that initially involved considerable input from NGOs, both in its pilot implementation and in the methodology for scaling up, and which has been scaled up at considerable pace.

### Health systems as complex systems: Conceptualising change

The overall ART programme, as it operates in health facilities in the Western Cape, can be conceptualised as a complex adaptive system (CAS). This dynamic view of a system as non-linear has become a widely applied framework for analysing health systems. It brings together a number of useful ways of analysing complex systems, with an approach focused on identifying patterns that emerge from the interrelationships and interactions of the constituent and interdependent parts [15, 16]. As such, health systems as complex systems are understood to be constantly changing, yet the outcomes of change are unpredictable, with the possibility of positive as well as negative unintended consequences [17, 18]. The CAS approach conceptualises distinctive and relevant properties of the system that have bearing on the nature of change, such as self-organisation, feedback loops, path dependency, emergent behaviour, and time delays in outcomes [16, 18, 19].

It is thus argued that the CAS lens is valuable for forestalling unintended negative consequences of a new or existing policy implementation, and for identifying positive synergies that could be enhanced [20]. On the one hand, an intended change in the form of an intervention will clearly affect the complex system and can have system-wide effects; on the other hand, the nature of the system in turn will affect the implementation of an intervention [18]. Dattée and Barlow [21] point out that complex systems are likely to be made up of structures at different scales, requiring a ‘whole systems’ perspective and the consideration of change across levels. Such a ‘systemic’ view might indicate that a more radical restructuring is necessary of the whole system and its core functions in order for a desired reform across the system to be achieved.

Whilst the dynamic nature of health systems as CAS could be seen to facilitate a responsiveness to change and policy directives [17], inertia and interdependencies in a complex system could also slow the pace of change [18, 21]. Furthermore, health systems are not only constituted from technical components, but also have distinctive histories, organisational cultures, differing governance and authority structures, and embedded power relations. An appreciation of these aspects and the interconnections of ‘software’ components (such as skills, leadership, values, and relationships), as well as the functional building blocks or ‘hardware’ [22], are all key to understanding processes of and responses to change [22, 23]. Moreover, the broader societal and political contexts in which health systems are embedded are themselves complex [22, 24]. These environments shape the ideological drivers of health policy and add political imperatives and cultural inflections to the framing of problems and to the shape and pace of change [25].

If we consider planned change in the form of new policy or protocols, both the internal characteristics of the health system as well as the surrounding context are thus important in order to reach an understanding of the factors that might inhibit or enable the acceptance and implementation of the desired change. These factors might include local facility-level capacities, buy-in from leaders and frontline staff, the degree of alignment with the ethos of care and the organisation of services, and the effect on existing workloads. Gilson et al. [23] argue that ‘everyday resilience’ in a health system is central for enabling positive responses to ongoing requirements for change. They contend that there are limits to the amount and pace of change that is feasible: constant requests for change brings strain to healthcare workers. Moreover, it cannot be assumed that a system will indefinitely absorb change and generate positive adjustments; adequate investments are also key, such as increased resourcing and staffing levels [23, 26, 27]. In this regard, policy rhetoric might inflate expectations by overstating the extent of whole system change that is possible, or overestimating the desirable speed of change [21].

Swanson et al. [19] draw on systems thinking to propose strategies that can enable system-wide transformation in a health system, which they argue is necessary to achieve more efficient use of limited resources and simultaneously achieve positive health outcomes. They identify three overarching principles: collaborative working across the system at intra- and inter-organisational levels; transformational leadership by individuals with the foresight to put system-wide benefits above personal and organisational interests; and a culture of continuous and iterative learning in the health system that recognises changing contexts and identifies and learns from new challenges. Drawing on a study in the Western Cape, Gilson et al. [28] argue that positive adaptive strategies depend on cognitive and behavioural factors, in particular whether actors down the levels of management can make sense of the requests for change and are willing to use their ‘discretionary power’ to implement it. An important governance objective for health systems is that they operate as ‘learning organisations’, responsive to their complex adaptive nature [19, 23, 29].

### Scaling up and innovation

The growing scholarship in recent years concerned with the ‘scaling up’ of interventions in health systems draws upon systems thinking approaches. A concern with scaling up is underpinned by the argument that achievement of the sustainable development goals requires a shift beyond small pilots to significant change [30, 31]. ‘Scaling up’ as a concept has been used to refer to an extension of the geographic reach and/or scope and coverage of an intervention [30, 32], as well as to the processes and capacities and resources required to achieve such expansion [32]. Key issues related to scaling up have been identified as the overall costs; the constraints to processes of scaling up; quality and equity considerations; and service-delivery issues [31, 32].

Attention to the appropriate pace of change and monitoring to ensure that intended effects (or at least positive effects) occur, becomes particularly important when scaling up change across a health system beyond a small, controlled pilot intervention. In this regard, systems thinking is considered a valuable conceptual tool to guide and achieve positive whole systems change at scale [17, 30]. This approach is linked to the principle of ‘learning by doing’ [33], including iterative monitoring to assess for negative consequences and difficult trade-offs. The perspective brought by a CAS approach can thus increase the likelihood of sustainable outcomes from scaling up.

The literature on scaling up refers to ‘innovations’ that might be identified as promising initiatives. An interest in innovation in health predates the greater prioritisation of scaling up, and spans high-income and low-income settings. There are, however, common themes, and complex systems thinking is relevant for anticipating and monitoring unintended outcomes [34]. An innovation in the health system might be a ‘grassroots’ response to an emergent problem, and involve an organisational change or a technical solution [35]. If such innovation then suggests positive benefits or productive ways of addressing a challenge, it might be identified at higher levels and formulated into an initiative for wider dissemination in the system.

Scholars have identified stages of innovation as applied to healthcare, starting from the identification and framing of the problem requiring a response, through to the wider adoption of the innovation and its diffusion and dissemination [35, 36]. The framing of the problem is influential with respect to emergent narratives about the success of an innovation. This can influence the degree of uptake of the innovation, if it has resonance with influential stakeholders [35]. In this regard, Denis et al. [36] examine the diffusion patterns associated with complex innovations in healthcare and conclude that equally promising innovations do not necessarily share the same level of success. Those who have a stake in the innovation, engage in weighing up the risks and benefits of wider implementation. Such pragmatic calculations can end up being more important than evidence about desirable outcomes. Indeed, diffusion might occur whilst evidence is still emerging, if the innovation seems to confirm common sense or addresses an obvious or urgent problem. Thus, as with scaling up, the success of a process of institutionalisation of an innovation is also dependent on political factors, the broader health systems environment, and the assessments of key actors. Furthermore, there are arguments in favour of inclusive processes of innovation that prioritise whether innovation meets democratising goals, with attention to ensuring that the direction of change is agreed by a wide constituency and that the distribution of benefits ensures that marginalised people are not left behind [37, 38].

Whilst the academic literature on innovation in health systems emphasises context and complexity, political rhetoric might not appreciate such nuance. A discourse of innovation within policy circles might do political work as a trope, implying a singular technological solution to problems, which consequently can belie the underlying complexities that need to be taken into account for sustained and positive change to be achieved. A ‘quick fix’ view of innovations also encourages singular indicators for monitoring the outcomes of innovation, with a focus on quantitative measures. Such indicators can isolate a focal point in a system and pay less attention to processes of change and the ‘software’ dimensions of health systems.

In South Africa, the health system has undergone significant restructuring since the regime change in 1994, involving frequent directives for change, and requiring a degree of ‘everyday resilience’ in the health system to adapt to new ‘standard operating procedures’ (SOPs) and priorities [28, 29]. Within an historically hierarchical organisational culture, this requires shifts to a flexible and responsive style from middle-level managers in district sub-structures in order to mobilise others to embrace change [29]. The language of innovation has also been evident in the search by policy-makers for solutions to identified problems related to HIV care, and more broadly in the existence of ‘social innovation’ awards for health. It is also the language that has currency for the way in which pilot initiatives by NGOs are discussed. This was the context in which we undertook the evaluation of the particular innovation that forms our empirical case study. We consider these broader questions related to complex systems and the consequences of introduced change, with reference to innovation and processes of scaling up in public sector health systems.

## Methods

The overall objective of the qualitative and quantitative components of the adherence club evaluation was to describe the health outcomes for patients accessing clubs, and to evaluate how the model of care performed at scale. The qualitative component that we conducted focused on a health systems analysis with the aim of describing and analysing the core elements necessary for rolling out the club model to new clinics, and for scaling up the number of clubs within any particular facility. We conducted the health system study in twelve facilities in the Cape Town metropolitan area, all of which had also been included in the epidemiological analysis. None of us conducting the health system study was involved in the design of the original club model, or in the roll out of clubs as a service delivery intervention in facilities across Cape Town.

We used a combination of methods, starting with observation in the clubs in all the facilities. Interviews were conducted within facilities with a patient receiving ART in an adherence club. We also conducted interviews with as many of the identified core cadres of staff involved in clubs as available, namely: a nurse, doctor, HIV counsellor, pharmacist, pharmacy assistant, and data clerk. We also interviewed facility managers and the ARV programme heads where possible, and the identified club coordinator. At the substructure level we conducted interviews with HAST (HIV, AIDS, STI, TB programme) medical officers in whose jurisdiction these facilities fell. We used a snow-balling approach to identify other stakeholders from the club steering committee, key NGOs, and senior WCDoH and CCTDoH policy-makers and managers. In total, we conducted 45 interviews with a range of different stakeholders, collecting different perspectives on clubs and the core components of the model. The interviews also focused on the initiation and scaling up of clubs, and reflection on the enabling and inhibiting factors, as well as the challenges of the process. We collected and reviewed key adherence club documents and routine monitoring data, and relevant provincial and CCTDoH policies. To capture the background to the initial club pilot, we conducted a workshop with a group of MSF staff, using an innovation histories approach [39].

Drawing on thematic analysis of the interviews, our observations and the document review, we mapped the system components and functions of the club model. We decided to adopt a CAS framework to inform our health systems evaluation of the club model, and to analyse patterns that we identified as emerging in the scaling up process, from a triangulation of all the findings. However, our analysis also draws more widely on literature on scaling up, organisational change and innovation, and anthropological perspectives on health in the wider context of development and the political economy of change. Our preliminary analysis was presented to a meeting convened at the WCDoH, in August 2016. This included key stakeholders and several of our respondents. A full report was produced for the CCTDoH and the WCDoH [40], and comment was provided on a draft version by key stakeholders to correct inaccuracies. This iterative consultation provided further insight into the process of scaling up and institutionalisation of the model, as well as thinking on future challenges and opportunities.

## Results

### The history of the innovation and methodology for scaling up

The innovation history workshop (see Table 1 for a summary of the innovation timeline for clubs) conducted with MSF staff indicated that in 2007 the NGO began piloting, in Khayelitsha, Cape Town, a model of differentiated care in a state facility that it supported. The model aimed to identify and support a category of ‘stable patients’ from amongst those on ART, by establishing ART adherence clubs. MSF has pioneered various models for decentralised and community-based distribution of ART in other African settings, such as community adherence groups [41–43]. The club model grew out of these experiences but appeared to have diverged somewhat from the earlier ‘support group’ ideas and dimensions of adherence counselling and peer support, focusing more on convenience for those with HIV. The original MSF idea of a club in Khayelitsha was reported by MSF staff to have come from the concept of an airline loyalty club, in that the model incorporated the principle that membership required certain criteria to be met in an ongoing way. Thus a person would lose club membership for missing a medication collection. An idea of club members as “VIP patients” was reported by MSF staff to have emerged at this time, implying that people were earning membership and a streamlined service as a reward for adherence. This term lingered amongst staff in a few of the clinics where we observed.

**Table 1:** Innovation timeline for the history of clubs and scaling up

Period	Development of adherence club model	Context
Pre-adherence clubs		
2000–2004	Prior to government roll out in 2004, MSF provided some ART in Khayelitsha, Cape Town.	HIV + ve patients started on ART in Cape Town.
2005-	Initiatives were introduced exploring models of service delivery to decongest facilities and streamline treatment in Cape Town area.	As increasing number of HIV patients received care, key stakeholders considered the decentralization of HIV services to decongest health facilities.
Post-adherence clubs		
2007	Stable patients moved to adherence clubs in 1 facility in Khayelitsha.	MSF has pioneered various models for decentralised and community-based distribution of ART in other African settings, such as community adherence groups
2010	MSF began discussions with the WCDoH and CCTDoH to adopt the model in Cape Town.	Funding for scaling up health innovations came from the Institute for Healthcare Improvement (IHI) which allowed the scale up of the club model.
2011	The roll-out of the model was instituted in facilities managed by both WCDoH and CCTDoH. The club model became instituted as policy.	The CCTDoH provided external support to the roll-out through central health staff. Likewise, the WCDoH gave this role to HAST medical officers in the substructures.
2012	Community clubs, youth clubs started to emerge.	The club initiative won a platinum award from the Impumelelo Social Innovations Centre. Over 15% of people on ART in Cape Town were part of clubs.
2012-	The club model is expanded to include co-morbidity clubs, family clubs and male clubs.	Emerging evidence suggest that stable patients on long-term ART can safely be offered differentiated care options.

A senior manager in the CCTDoH recalled that, from the mid-2000s, several other initiatives had been tried across the metropole, exploring models of service delivery to decongest facilities and streamline treatment. One system involved different coloured stickers on folders in an attempt to indicate different streams of care.^3^ The possibility to scale up the MSF club model within the state services she ascribed to a serendipitous convergence, in 2010, of three factors: emerging findings from MSF’s pilot club initiative; pressure to address the problem of facility congestion; and some funding that become available for scaling up. The model attracted attention and, in 2010, MSF began discussions with the WCDoH and CCTDoH to adopt the model. A collaborative workshop was held in December 2010.

Funding for scaling up came from a grant to another international NGO, the Institute for Healthcare Improvement (IHI). IHI pursued the opportunity to implement a particular methodology for scaling up health innovations that involved regular external support and trouble-shooting in facilities where implementation occurred, as well as workshops for learning across facilities [44]. The interest of the WCDoH and CCTDoH in the MSF clubs provided an opportune case study of an innovation that seemed ripe for scaling up. Thus, a limited number of facilities under the management of the CCTDoH as well as the WCDoH were selected for the first wave of roll-out. The roll-out was initiated in 2011, a steering committee (hereafter ‘committee’) was established,^4^ mentoring of facility staff was instituted, and the club model became policy in the WCDoH. The government framing of the programme did not emphasise support for disease self-management as a key element of the club experience. The poster in clinics explaining clubs (and the criteria for eligibility) emphasised the convenience: “Fast. Friendly. 2 months [sic] supply of ARVs”. It posed the question: “Are you tired of waiting in long queues every month?”

An initial target was set by the committee for 30% of those on ART to be put into clubs. A committee member admitted that this figure was an estimate, a “thumbsuck”, but that this target was considered low, and deliberately so. MSF staff identified the adoption of the clubs as policy for the ART programme by the WCDoH as a key tipping point in their advocacy efforts to achieve the scaling up of the model. The CCTDoH was particularly active in providing dedicated support to the selected facilities under their remit, through allocating external mentors from amongst the central CCTDoH health staff. The WCDoH gave this role to HAST Medical Officers in the health substructures. A CCTDoH programme manager indicated that the idea had been to start slowly, and thus the roll-out occurred over several years with 3 groups of facilities identified for 3 waves of extending the initiative.

In 2012, the club initiative won a platinum award from the Impumelelo Social Innovations Centre. A publication in 2013 [45] laid out the principles and procedures for clubs and reported on the early experience of the roll-out. By the end of 2012, there were over 600 clubs across the Cape metropole, involving 16,000 people receiving ART through clubs. This constituted about 15% of people on ART in Cape Town. By the end of March 2016, approximately 32% of people in the whole ART programme (42,600 of an overall total of 142,000 ART patients) in the Cape Metropole were in a club. The target for recruitment to clubs had shifted to 50%. However, the picture that we found was mixed across the facilities, both CCTDoH and WCDOH, with the numbers ranging from below 10% to nearly 60% of people on ART in clubs. In one large facility, 90 clubs were running. There was talk of a further target as high as 70%. However, there had been no formal assessment overall of how many people on ART could be considered ‘stable’. At the time of our study, research on the club model had been limited to evidence from group-level monitoring data [41]; the findings from the original pilot study in Khayelitsha [46], which showed that the model of care was associated with high levels of retention-in-care and viral load (VL) suppression; a study of the cost-effectiveness of the model [47]; and outcomes from a single facility [12, 13].

In June 2016, just as our qualitative fieldwork was ending, the first results of the long-awaited epidemiological analysis of clubs, the quantitative dimension of the overall evaluation, emerged. It assessed retention-in-care and VL suppression for people in clubs in a sample of facilities, against outcomes for the routine service of the ART programme in the Western Cape. Results were presented at the International AIDS conference in July 2016, and initial data were positive with respect to the clinical outcomes from the adherence clubs. For the 3216 adults sampled, retention was 95.2% (95% CI, 94.0–96.4) at 12 months and 89.3% (95% CI, 87.1–91.4) at 24 months after club enrolment. In the 13 months prior to analysis closure, 88.1% of patients had viral load assessments and of those, 97.2% (95% CI, 96.5–97.8) of patients were virally suppressed. Significantly, risk of Lost To Follow Up (LTFU) from clubs was higher in younger patients and in patients accessing ART from facilities with larger ART cohorts. Risk of viral rebound was higher in younger patients, those that had been on ART for longer, and patients that had never sent a ‘buddy’^5^ to collect their medication [48]. This represented the first analysis with reporting of patient outcomes, after health authorities scaled up a differentiated care model across an entire district in a high burden setting. The quantitative analysis thus provided substantial reassurance that stable patients on long-term ART can safely be offered differentiated care.

### The framing of the problem, and the drivers of innovation

The interviews conducted with a range of stakeholders in the WCDoH and the CCTDoH, who had been involved in the club initiative, revealed different perspectives and framings of the problem that was seen to have necessitated innovation. All the interviewees agreed that the initial thrust for innovation was from the service side, and related to the concern that clinicians were overwhelmed by the sheer numbers of HIV positive people in facilities. In some accounts, this “congestion” problem was then linked to patient safety concerns, in that overcrowded spaces potentially introduced unnecessary exposure to infections such as multi-drug resistant tuberculosis. Some policy-makers mentioned that sub-optimal retention-in-care was a related concern, which a streamlined service could potentially mitigate. It is unsurprising that declines in retention-in-care and concerns about the spread of resistant tuberculosis featured in accounts; these outcomes challenge the narrative of success of the national HIV programme. On balance, however, the problem of coping with large numbers of HIV positive people on treatment in facilities was seen as the most prominent driver of change from the service side. This problem of congestion was presented by the policy-makers as one that was exerting strong pressure on the system in an ongoing way, necessitating action of some kind. Several respondents mentioned the looming adoption of the WHO guidelines that would necessitate more people being enrolled in the ART programme.

The issue of quality of clinical care emerged as a contentious dimension of the decision-making to institute clubs. On the one hand, some interviewees cited declining care as a reason for clubs, in that overloaded clinicians were thought to provide care of lesser quality. On the other hand, medical officers reflected that from the outset there were concerns that clubs could have negative impacts clinically, given the less frequent attendance, clinician contact and blood monitoring, as well the reliance on CHWs for running clubs and mediating that contact.

A further political pressure driving the club initiative related to concerns expressed by senior policy-makers as an “equity” issue; the HIV programme, in the words of one, has constituted a “Rolls Royce” programme, with greater access to resources than initiatives for other diseases. The discourse of equity holds a powerful resonance in South African political life, given the country’s history of institutionalised inequalities. The 2030 health strategy for the Province is committed to improving care for people with non-communicable disease (NCD), also a high disease burden gaining increasing attention in South Africa [7]. This commitment was seen by some interviewees to require a greater sharing of resources and learning from HIV care with programmes for NCD, and even integration of care into a chronic disease stream under the National Department of Health’s ‘Ideal Clinic’ initiative. One means to achieve this has been through the pursuit of routes to more efficient uses of HIV resources. The hope was that this could partly be delivered through task-shifting and streamlining in a differentiated care approach.

The degree to which evidence informed the decision to adopt the club model as policy, was reflected upon spontaneously by interviewees. A key decision-maker in the WCDoH mentioned that the relationship with NGOs such as MSF provided the department with the experience from an existing innovation; the department itself did not have the capacity to conduct pilots to inform a scaling up of service innovations, such as the extension of clubs. This extension was thus not formulated as research, but as an innovation in service delivery. The initial pilot data that was emerging from Khayelitsha, was then boosted by positive feedback once roll-out began. This was gathered from HAST medical officers doing routine programme visits and clinical audits. As a policy-maker put it: “We could see the benefits”. However, a doctor who had worked in a large, high burden facility expressed a misgiving that was echoed by other clinicians: that the pressure of congestion had caused pragmatism to prevail, with the innovation having been adopted too early and before a wide-scale evaluation had occurred. In this regard, some anxiety was evident on the part of key stakeholders regarding the pending results of the epidemiological analysis, given that viral load and retention-in-care could be seen as proxy indicators of quality of clinical care and the functioning of the club system. Significantly, reflecting on the fact that the evaluation was still pending but the club scale up was in full swing, a CCTDoH policy-maker reflected that “we can’t go back now”. This indicated a pragmatic acceptance that a point of no return had already been reached with the diffusion of clubs across the facilities. In her view and that of others, it would have been very hard to undo the club intervention due to the fact that facility staff simply would not cope with the numbers in the routine service. Patients would also not be willing to give up the convenience. In our later interactions with policy-makers after the release of the promising epidemiological results in June 2016, there was relief expressed that the “risk” had paid off to push ahead with the scaling up of the club innovation. The rapid scaling up of clubs typifies a circumstance where a service intervention was implemented in pragmatic circumstance where there was a juggernaut of change and pressure on policy-makers to provide ‘solutions’ for challenges in service delivery that could quickly translate into practice. Our evaluation of the scaling up of clubs was an example of the kind of collaboration between policy-makers and researchers that is not uncommon in contexts like South Africa [25].

Consideration of the experience of HIV positive people on the demand-side of the ART programme also featured as a secondary driver of innovation. Facility-level clinical staff did reflect on the value of clubs as a tool for motivating adherence, since, in the experience of staff, people did not wish to lose club benefits. Interviews that we conducted with people in ART clubs confirmed the benefits of club membership to them in terms of convenience, in particular related to bypassing facility queues and enabling a quick appointment. Most clubs were run early in the morning, allowing those in clubs to get to work for the day. For example, one man had asked to be put in a club when he heard that they existed in his facility, noting that the biggest difference from the routine service was the reduced time. He could leave the clinic in 1 hour, whereas he used to get back home in the afternoon. He also had been able to get admission to the same club as his wife, and they could make use of the ‘buddy’ system in clubs to stand in for each other in medication collection. His sister-in-law had recently requested also to be put in their club. Whilst his account emphasised the convenience of a quick service, he also spoke of the longstanding counsellor in the facility who offered support, and the support he drew from being in the same club as his wife. A man at another clinic emphasised the same advantage of being in a club with his wife. Whilst his wife chatted to others at the club, he did not reach out in this way.

### Key components of the model: Early evolution and adaptation for scaling up

From the outset, clubs were set up to provide ART support to groups of approximately 30 people per club, who would meet and receive medication every 8 weeks (as opposed to monthly). Some of the key components of the clubs evolved within the MSF pilot innovation and were features of the original model, but further adaptations emerged to meet the early requirements for scaling up and to address problems that arose as the scale up of clubs intensified. Key managerial processes (such as an organogram of staff roles^6^) and monitoring tools were discussed under the guidance of the steering committee, which developed SOPs in 2011. The systemisation of club protocols aimed to provide guidelines for the functioning of particular sub-components of the club system and were intended to enable the institutionalisation of differentiated care. The process of scaling up was a catalyst for such consolidation of existing procedures. Government stakeholders reflected that provincial buy-in enabled leaps in the organisation of clubs, which greatly enabled the degree of scale that became possible to achieve.

The systematisation of the criteria for the identification of ‘stable patients’ for placement into clubs was crucial for the initial thrust to set up the intervention in facilities. The MSF criteria were adopted at the outset, but as the scaling up progressed, the entry criteria were lowered to enable rising recruitment targets. For example, by the time of our fieldwork, the length of time a person had to be adherent on ART had decreased from 18 to 6 months. Only annual blood and clinical visits were stipulated, however the requirement for recruitment into a club to be authorised by a clinician had not changed.

In the MSF pilot, task shifting in the club model involved the shift of the management of those in clubs to CHWs in the form of HIV counsellors. This cadre also existed in state HIV clinics not supported by MSF and counsellors were now given the additional role of engaging in the face-to-face patient contact in clubs. A key part of the clubs was documenting who attended and collected the medication. In the pilot, this had been done by putting folder stickers onto pieces of paper, but a standard hardcopy register was now formalised. Facility folders were no longer drawn for clubs.^7^ Registers recorded the names of people in a club, whether medication was collected, a weight, and whether any concerns arose regarding health. At the beginning of the scaling up there was no capture of register data, but a system for monitoring was developed to enter information from club visits into the HMIS and to reduce the chances of spurious LTFU, whilst providing a back-up for noting missed appointments. As the number of clubs per facility rose, the club rota became more complicated and an electronic scheduling tool was developed.

Pre-packing of customised medication packages is a core feature of clubs and this was initially done by the respective facility pharmacies, as in the pilot. This function was taken over by an existing central chronic dispensing unit (CDU), operated on tender by a private logistics company on behalf of the WCDoH. Most of our interviewees saw this shift as one of the main contributors to escalation of the club model. A key manager in the provincial pharmacy services indicated that the CDU, as an innovation, was not specifically developed for clubs but was conveniently harnessed for use by clubs. The clubs also utilise a system whereby medication is provided for 2 months. This possibility also predated the clubs. Individual clinicians had also, for years, informally provided 4 months of drugs over Christmas holidays. This was made official in clubs, referred to as “the jump”. A stamp with the HIV core regimen was produced to ease the time-consuming hand-writing of renewal scripts. A doctor also developed an electronic scripting tool but it has not become official for use in clubs.

A senior CCTDoH manager expressed surprise regarding the degree to which different interpretations of clubs became evident as the model disseminated across facilities. She also described how one busy facility, not identified for the initial roll-out, ‘leaped-frogged’ the plans and spontaneously adopted the club model through self-organisation, when staff heard about the innovation and felt it would be of value.

### Factors that enabled the scaling up: Stakeholder perspectives

Our analysis suggests that progress in extension of the model across facilities, and in scaling up of the number of clubs in particular facilities, could be ascribed to political factors and leadership at all levels of the health system. The intervention chimed with an external political moment when there was pressure to identify efficiencies in the resourcing of the HIV programme. Within the public sector, there was clearly an appetite for finding ways to cope effectively with “congestion”. This was synergistic with the concretisation of the club innovation and harnessed political will for scaling up. For decision-makers, the benefits of progressing with the adoption were assessed to exceed possible risks and concerns. There were influential people in the committee who heavily influenced this and drove the development of the clubs.

In facilities, it was also evident that relationships and leadership contributed to the way in which scaling up progressed, in particular with respect to the extent of commitment to the idea of clubs. Similarly, interviewees mentioned the importance of a “champion” for clubs amongst key staff members and driving the recruitment in a facility. An identified nurse champion was deployed at a higher level to visit different CCTDoH facilities to enthuse staff and drive recruitment for the 50% target. A senior CCTDoH manager reflected that she had not appreciated in advance the extent to which champions at facilities, individuals rather than a staff cadre, would drive the roll-out of the intervention. This overall view is in keeping with CAS thinking regarding the importance of networks and hubs in influencing systems change.

The extent of close support at facility-level from central CCTDoH and WCDoH managers and the designated mentoring was cited as important to buy-in and to enhancing a feeling that clubs “made sense”. Certain key clinics as ‘early adopters’ influenced other clinics. The IHI methodology for scaling up received positive assessments. The dedicated committee was seen as key to the scaling up, and particularly in the way that it also enabled coordinated working across the somewhat fragmented service platform, cementing collaborative relationships between CCTDoH and WCDoH. Similarly, meetings occurred between the senior management and the next sub-level. The regular facility-wide workshops were also seen to have contributed to driving the process. The structures thus set up, specifically to shepherd the scaling up process, enabled key relationships and dedicated focus. They also enabled appropriate provincial directives, necessary for clubs to operate more effectively at scale. As one interviewee put it, the presence of senior managers enabled the committee to “unlock problems quickly”.

### Factors that inhibited scaling up: Stakeholder perspectives

At a pragmatic and individual level, an inhibiting factor for scaling up in facilities related to the energy required to initiate change and maintain it, it in the midst of pressure from other service delivery priorities and other directives. As one clinician put it, “it is pretty hard for people to be pushing a new system”. He reflected that ongoing “input” was needed so that the initiative did not “fall off the agenda”. Furthermore, concerns were evident (amongst some clinicians in particular) about the potential for clubs to compromise clinical care in the pursuit of other goals. These anxieties about a reversal of hard-won gains in HIV care seemed to underpin some resistance to clubs and to the pace of the scale up. One clinician expressed a stark opinion: “Should we destroy what we have built or transfer best practice over?”

Several interviewees reflected that with time the frequency of mentoring and workshops had reduced, as the number of facilities involved increased and meetings became logistically more difficult. The tapering of external support with subsequent waves of roll-out was seen by some as appropriate, as the model was considered to have “taken root” by the third wave. However, others felt it had reduced the drive for change and mutual learning. Indeed, the support had become more fragmented and the tight coordination that was remarked upon from the early waves of roll-out appears to have dissipated by the time of our study. A senior WCDoH official felt that this was leading to an “overreliance” on the HAST medical officers, commenting: “You cannot expect them to drive everything”. Indeed, HAST staff were also being pressed to drive ‘integrated’ service-delivery for NCDs.

A related factor was a lack of clarity regarding the ongoing role of the steering committee. Whilst the formalisation and commitment of the committee were remarked upon by most interviewees as a strong enabling factor, with “experts and enthusiasts around the table”, one senior committee member reflected that, with time, the committee had come to lack direction and the effort was “fizzling out” somewhat. This was also seen by this respondent to reflect a lack of strong WCDoH buy-in at the highest level, so that further efforts were not enabled. This was ascribed to concerns about whether the model actually did save money. There were strong feelings from some that a committee was still needed to address ongoing emerging challenges of scaling up. Another view was that the time had come for further decentralisation, with SOP “circulars” to facility managers and “capacitating the frontline”.

The fact that staff at facility-level did not always appear to appreciate the benefits of the clubs in their day-to-day work and, in reality, perceived clubs as bringing work, was seen as a significant inhibiting factor. The success of scaling up was articulated in terms of progress towards the percentage targets for recruitment, and most interviewees referred to a slowing down or “recruitment plateau” in many facilities. Support for clubs was drummed up with the rationale that they would decongest the routine HIV service, referred to informally as the “floor”. However, it appeared that healthcare workers were not experiencing this promised relief, ascribed in part to ongoing movement of newly-initiated people into the ART programme and rising HIV prevalence as people lived longer. Rather, not only did the recruitment of a person into a club involve more work in that consultation, but the actual running of clubs was also remarked upon as requiring immense logistical effort. Many interviewees, at all levels, admitted that the extent of the workload of clubs simply had not been appreciated in advance. This effort was becoming even greater with the push for more clubs per facility, as the level of organisational complexity increased correspondingly. A whole-systems view of the programme suggested to us that a negative feedback loop was operating, where progress in scaling up in a facility (and thus growth in the number of clubs), was increasing workload. This burden then acted back to influence staff behaviour by dis-incentivising active recruitment of even more people into clubs, and a consequent further increase in work. Such a loop acted to slow down further scaling up, and also progress to the recruitment targets.

Despite the evident workload associated with clubs, they were still not perceived as being core programme work. This had a negative effect on scaling up in that it was harder to motivate for a sharing of the effort involved, limiting the capacity for change. The committee was advocating for management to promote clubs as “the new normal”, business-as-usual to be shared by more staff, with certain days scheduled just for clubs. In some facilities, clubs were run as a separate entity with a small proportion of ART programme staff involved, whilst others did not see them as a central feature of HIV healthcare. We heard in some facilities of non-club staff resisting involvement in clubs, which were seen as “extra work”. A WCDoH manager admitted that this perception made it “tough to convince facilities”. The shouldering of the lion’s share of an expanding workload by a few (and sometimes, just one) increased the chance of burnout and diminished enthusiasm to drive further scaling up. Such burnout was particularly evident in interviews with counsellors, who were often given a disproportionate share of club responsibility. A de facto separation of “club” and “floor” efforts (exacerbated by the remote location of some clubs) could further result in less integrated functioning across the programme and a diminished appreciation of the position of clubs in the whole ART programme.

Whilst the committee had worked hard to systematise procedures for starting clubs, there had been less effort to formalise plans for addressing the organisational complexity that accompanied a large increase in the number of clubs in a facility. For example, the original club model specified who should be in a club team, in terms of the different staff cadres and tasks (although in practice there was considerable variation). What was not specified, however, was whether, as the number of clubs grew, teams should be replicated so as to manage a fixed number of clubs per team, or whether the single club team should be expanded and should manage all club members. It appeared that the spiralling level of organisational complexity had caught many off guard. A reactive working culture still appeared to be the norm, whereas it was fast becoming evident that the club model required advanced planning and proactive working if a large show was to remain on the road. Accounts of the early days of club initiation included stories of champions who single-handedly ran the clubs. However, a scaled up club system required broader teamwork, and sharing of learning to take account of staff changes. In one facility, a “champion” had retired. Her colleagues admitted that chaos has ensued, as others had scant knowledge of club logistics. The fragmented community-based service platform also accounted for unexpected staff changes amongst counsellors. They were contracted by NGOs which rotated staff between facilities and roles. This further impacted on the continuity of club skills. In a few facilities, the relationship between the NGO and management was fractious, as senior staff were seen as unsupportive of the work of counsellors.

On the logistical side, problems of capacity related to the orchestration of the CDU system and the pharmaceutical dimension of clubs emerged as the biggest cause of the kind of club “chaos” that could paralyse service delivery and increase the chance of inertia in the face of directives to scale up. Many of the interviewees who had witnessed the waves of scaling up identified the shift to the use of the CDU and the outsourcing of the medication packaging as the major organisational challenge that had been faced by clubs. There were accounts of technical errors on the part of the logistics company, especially initially. This coincided with a new service provider taking over the running of the CDU. The pharmacy component of clubs was complex, and we identified several weak points resulting in errors. For example, deadlines were set for scripts to be submitted six-monthly to the logistics company, and these had to be met for the packages to arrive for subsequent clubs. At the time of our study, hard copies of scripts were still required and basic issues such as computer literacy and broken printers were still causing hiccups in getting scripts in on time.

The question of resourcing was referred to repeatedly as an emerging challenge as the scale up of clubs was escalating. This related to inadequate staffing levels and also concerns about poor infrastructure, with tiny rooms and a dearth of off-site spaces. A WCDoH HAST manager saw this as inevitable but not insurmountable with better training: “There will always be teething problems when you have an NGO pilot that is taken over by a government and the NGO has much more support”. A senior CCTDoH manager, however, was robust in her opinion that challenges were emerging because extra resources were not being made available, as was clearly necessary as the scaling up progressed. She felt there was a misconception that clubs were a means to save time and resources, so that nothing extra would be needed. However, differentiated care in fact represented, in her view, not a more efficient, but a different way of working. In some instances, more complicated, unofficial procedures had become a norm in a facility,^8^ and there were points for “efficiencies” in clubs. However, overall, she reflected, there was a basic issue of needing more hands for service delivery, as well as allocation of more time for management. A CCTDoH manager echoed these concerns but reflected that any additional resources would prioritise a sicker category of patient. His assessment was that clubs would have to manage with limited staff and inadequate infrastructure, which would make the achievement of the targets very challenging.

Finally, as assessed from the accounts of clinical staff and the interviews that we conducted with patients in the clubs, there did not appear to have been significant resistance to the institution of clubs or to the scaling up, although people complained about logistical incompetency in the system. One man had time to be interviewed because on that day he was also collecting his wife’s medication, and a scripting error had meant that her medication package was incomplete. At another clinic, a man spoke of changes in the club logistics after retirement of the nurse who had driven clubs. Now they saw different staff members and the continuity of care had been lost. One physician did reflect that, in his experience, patients had to be convinced that leaving routine care for a club was a good idea. Apparently, some were reluctant to give up one-to-one care, and with physicians with whom they had longstanding relationships. Indeed, we observed that clubs afforded scant privacy of interaction, although in theory people were able to request time, including with a nurse. For some people, the facility was actually easier to reach than the club venue, which was a disincentive to join. There were accounts from staff of considerable unhappiness from those “kicked out” of clubs because of missed collections. Some clinicians felt that recruitment into clubs had not been dealt with fairly, and a more orderly recruitment process would have allowed the patients with the longest clinic attendance, first entry into clubs. One person in a club noted that the non-club patients sitting in the corridor would speak out at the club patients as they perceived them to be getting preferential treatment. However, he put this down to lack of general knowledge about clubs so that these people perhaps thought that club members were regular clinic attendees just trying to get around the system.

### The forty club hurdle and complex system failures

Our evaluation occurred at a point when the scale up of the number of clubs in facilities to meet targets had escalated the degree of organisational complexity of clubs. Yet the level of support had dropped off. It was evident that the scaling up was revealing vulnerabilities in the club system that required further adaptation. Our study indicated that a negative ‘tipping point’ was evident in facilities that amounted to a breaking point with respect to logistics. We identified this as the ‘forty club hurdle’, a point at which logistical problems in a facility and failures in the complex system seemed more likely to occur. Most facilities had increased the number of clubs by allocating a club to a day of the week. The critical tipping point became evident when all the days were used over the 2 month window between club dates. Thus, when a facility reached 40 clubs, it became necessary to schedule 2 clubs per day. At this point, the level of complexity requires seamless logistical preparation. Given the interconnection of the different components required for adequate functioning of clubs, an error in one component was likely to have a domino effect across the whole club system. This analysis thus revealed very clearly that change can have unintended negative consequences, and the vulnerabilities in the system might only become evident once the scale of change exceeds the capacity for positive adjustments.

Such potential unravelling of the system was evident at several levels. Firstly, the vulnerabilities in the pharmaceutical delivery system came to the fore if the volume of scripting exceeded capacity. In some facilities, it had become all too commonplace for drug boxes not to be delivered because the scripting deadlines for clubs had not been met. In one facility, people were told that they might sometimes have to queue at the pharmacy with handwritten scripts; the medication packages were a “luxury” that could not always be provided. In some facilities, we witnessed maladaptive strategies, such as “spares boxes” of drugs being kept in the club room, which consisted of uncollected medication packages. Rather than returning these to the pharmacy, these drugs were used as a surplus for issuing medication to other individuals when errors occurred. Secondly, we witnessed facilities so overwhelmed by the numbers of club patients that the procedure for taking people out of clubs if they failed to attend, was simply not operating. The capacity did not exist to adjust the scripting cycle and care-plan for an individual. Finally, the scheduling of 2 clubs on a day created a timing issue. Many facilities scheduled the second club later, which compromised a key club benefit. As a result, we observed that most of those allocated a late slot came early anyway, creating logistical problems.

Our interviewees were largely aware of these problems. A committee member reflected: “We will need to do something different to get to 70 percent”. Another remarked that “growth is easy, maintenance is difficult”. Clinicians in particular were anxious about achieving tighter clinical governance in clubs. However, in a pragmatic, utilitarian vein there was a sense that, on balance, the initiative was sustainable. This sense was increased by the release of the epidemiological evaluation, with one stakeholder remarking that, despite all the system failures, the clubs must be doing something right. A CCTDoH manager commented that the momentum for clubs was really increasing and that the impact of clubs would soon become evident in terms of the long-desired “decongestion”.

### Future adaptation of the model and the emergence of new innovation options

In many regards, this optimism for clubs reflects the fact that the club model has been rich in ongoing innovation, both from a committed group of programme managers, and from the grassroots. In addition to the many adaptations in response to emerging problems, the model has stimulated further innovation on the back of its success. There has also been a diffusion of components of the club model to the general clinic systems, such as in the piloting of an appointment system. The model has also been discussed for transfer to NCD care. The club model has been adopted as national policy for South Africa [49]. The IHI methodology for scaling up has been considered for a new approach to the ‘Risk of Treatment Failure’ component of the ART programme. The scaled up club system also provided an organisational vehicle for facilitating the implementation of other service initiatives, such as the provision of tuberculosis prophylaxis.

Ideas regarding future adaptations of the club model (see Table 2 for a summary of some existing and future innovations) included integration of the pharmacies’ stock-monitoring systems and the CDU, to monitor non-collection and provide back-up of the register system. An official system for electronic scripting was a priority for all interviewees. There is clearly scope to explore electronic submission of scripts to CDU, although infrastructure and skills in facilities are lacking. The WCDoH managers indicated that they were engaged in advocacy to increase scripting requirements from six-monthly to annually. A new cadre of low-level pharmacy workers has also been approved to address legal grey areas in terms of dispensing off-site. However, enthusiasm for four-monthly clubs was not evident at policy level. A MSF advocate was of the view that the extent of clinical monitoring could be further reduced, the register simplified, and data capture streamlined. With the changes introduced by clubs, a separation of the different functions of service delivery was evident, in particular of clinical care and medicines dispensing, as suggested as a possible adaption for Southern African health systems facing the scaling up of ART with limited resources [50].

**Table 2:** Possibilities for further innovation

Existing innovation that have developed in the club model	Purpose	Transferability to other chronic diseases	Anticipated new challenges	Potential future innovation
Services Delivery				
Specialized clubs	Address special needs of patients (eg, families and adolescents)	Yes, transferable in some cases (eg. diabetes)	Other needs that could be addressed with separate clubs	Clubs for migrants, older people, sex workers etc.
Quick-Pick-UP (QPUP)	Lengthy times at facilities and desire for even greater convenience and collection flexibility	Yes, transferable	Other pharmacy pick up models	Private pharmacies, dispensing machines, home delivery options, use of unique patient identifiers
Community clubs	Improve community involvement, reduce stigma	Yes, transferable		Decentralised community medication distribution
Recruitment champion	Number of club members plateauing and reluctance to recruit	Yes, transferable	Club numbers vary across facilities	Roving club recruiter/mentors
Health workforce				
Task shifting of clinical care to HIV counsellors / CHWs extended to include moves for low-level pharmacy assistants	Initial task-shifting to CHWs because clinicians overburdened with stable patients; now need to ensure legal dispensing of medication and to relieve pharmacy staff.	Yes, transferable	Constant updates on clinical care issues needed	Creative training / mentoring of HIV counsellors / CHWs and of staff dispensing medication
Medical Products				
The ‘jump’ (the seasonal shift to a 4 month drug dispensing cycle over Christmas)	Seasonal migration of patients	Yes, transferable	Client push for 4 monthly visits/refills to become the norm	Annual script
Electronic scripting for processing the volume of scripts for CDU	Time taken with manual scripting	Yes, transferable	Harmonising of the electronic scripting with the official Western Cape systems; Capacity and resources for a paperless system	Electronic transfers of scripts from pharmacies to CDU and integration of CDU system with pharmacy stocktaking systems

This table describes (1) existing innovations that have developed around the core club concept, (2) the purpose for which they were developed, (3) transferability to care for other chronic dieases, (4) anticipated new challenges that could emerge, and (5) likely future possibilities for further innovations

A WCDoH policy-maker, reflecting on the looming adoption of ‘test and treat’, commented: “We will have to find options”. The club model had already stimulated other options. In Khayelitsha, MSF has run community clubs in members’ homes. Specialised clubs have emerged to cater to specific gender- and age-related needs. Co-morbidity clubs have spread for HIV positive people with NCD. Such people were initially excluded from club eligibility but now receive a blood pressure and glucose measurement at clubs. A more significant departure has gained ground: with quick-pick-up (QPUP), easy medication collection is the essence. In this development in the scaling up process, a complete departure is evident from the more sociological aims of support and counselling evident in the conceptualisation of early community adherence groups. The central idea has been reduced to the mechanics of medication dispensing. Pre-packaged medication is available in extended hours directly from the facility pharmacy. Some interviewees saw the CDU innovation as key to these spin-offs. However, it could also be viewed as having ossified into a form of path dependency. Indeed, departing from the CDU idea, the WCDoH has been investigating unique patient identification numbers which would operate platform-wide and could delink medication collection from a designated ‘home’ facility. There was also appetite amongst policy-makers to explore partnerships with private pharmacies as medication collection points,^9^ and for home delivery.

## Discussion

Our study took place when the last wave of roll-out of clubs across the Cape metropole facilities was virtually complete. However, the steering committee had significantly stepped up the scaling up of the club system within individual facilities, through targets requiring operationalisation of additional clubs. The scaling up process had thus spread across the geographic range and the scope of the initial pilot model. Our analysis revealed how a programme initially representing a simple, unitary system in terms of management and clinical governance (an ART clinic with a “floor” and club component), has evolved over a decade into a complex, differentiated care system. The ART programme included different streams for care, involving regular clubs, specialised clubs, a service for ‘Risk of Treatment Failure’, and new innovative options such as quick-pick-up. Services operated in different locations, with management and clinical governance spread over the whole system.

With respect to the clubs, what appeared to have been a partly pragmatic decision on the part of policy-makers to roll-out with early emerging evidence, was assessed to have paid off. People in clubs also expressed appreciation for the convenience and the early timings of clubs. Yet the operation of the club system itself had also become more complex, with many sub-system components that had to interconnect for optimal functioning of the service. The level of logistical complexity represented by the ‘forty club hurdle’ appeared, from a complex systems analysis, to constitute a negative ‘tipping point’ in many facilities: the capacity to compensate for errors (through positive and negative mechanisms of adaptation) was exceeded and a single organisational failure could decompensate the functioning of several related components, in a snowballing effect. It is also significant that the epidemiological analysis revealed that Lost To Follow Up was higher for people receiving ART from facilities with larger ART cohorts. Clubs as the “new normal” had not yet been adequately factored into management cultures, capacities, and human and material resourcing.

The SOPs for the club model outlined the functioning of clubs very proficiently, adequately informing a roll-out phase for starting clubs afresh in a facility, and detailing the methodology for supporting mutual learning across new facilities in waves of adoption. However, organisational protocols did not explicitly account for adaptations to consider the smooth functioning of the model at a significant scale, as more clubs were added per facility. Documentation had not evolved to produce an output informed significantly by what was happening ‘on the ground’ and to distinguish the distinctive requirements of ‘roll-out’ and ‘scale up’. The extent of creativity, solution-finding, and emergent spin-off innovations appeared impressive, but a systematic sharing of such learning across the service platform had also waned. The ‘innovators’ had an important ongoing role in pursuing new options and efficiencies, but the time was ripe for solid effort by ‘institutionalisers’. Hand-in-hand collaboration seemed necessary to address the challenges of significant scaling up, and to bed down the scaled up model, and ensure its sustainability in a challenging political environment with competing priorities within the ART programme and across the health system as a whole. A senior WCDoH policy-maker indicated that requests for information about clubs were coming in from other provinces, but the documentation required updating: “We need to reflect on the challenges as well”. This kind of synthesis of learning is vital to inform diffusion of the club model, both geographically, but also to other chronic disease groups.

Swanson et al. [19] emphasise collaborative working across a system, transformational leadership, and a culture of iterative learning, as key ingredients to enable positive transformational change across a system. Our findings concur with this emphasis and with literature on the importance of health systems operating as learning organisations [23, 26]. On balance, it appeared that ongoing management support through a steering committee-type body (with clear recourse to the most senior leadership), as well as structures down the management levels for supervision and mentoring, were important to sustain the system as it matured and the challenges of significant scaling up manifested. As the overall club system moved to higher percentages of enrolment, new ways of functioning were necessary to mitigate the danger of complex system failures. Our findings also concurred with observations regarding the importance of buy-in from middle-level management like HAST medical officers in order to sustain a ‘sense-making’ for change at the coalface and to drive iterative reflection [28, 29].

At a systems level, our findings revealed the importance of vigilance for the unintended consequences of planned change [17, 18], both to identify positive emergent behaviours, but also maladaptive responses and negative consequences of change. This monitoring seemed as important for the scaling up as it had been for the roll-out of clubs, bearing in mind also that some consequences of change can be delayed. We observed that, with a significant increase in the pace and scale of change, an individual drive to sustain and adapt to change was no longer sufficient. Increasingly teamwork was necessary, as well as a whole systems lens for appreciating the change required [21]. A WCDoH manager remarked that one disadvantage of a dedicated club-focused committee was that the “big picture” was more readily lost. If clubs were really to become “mainstream business” within the whole of the ART programme, a greater appreciation was needed of how clubs fitted in with the other differentiated options for care, with analysis of emerging trade-offs and the impact of clubs on the functioning and outcomes across the HIV clinical service.

Pania and Peters [17] argue that systems thinking can enable positive whole systems change at scale. We were not involved in the service delivery intervention and the planning of the roll-out of clubs. However, for our study of the scaling up of clubs, we adopted a CAS framework as an instructive approach. We observed, mapped and analysed the dynamic nature of change across the articulated components of a system, and sought the perspectives of different stakeholders on the process of change. We interviewed actors at different levels of the health system and from different staff cadres at facility level, enabling different views to emerge of the framing of the problem, the assessments of the benefits of change, and the factors which enabled and inhibited the process of scaling up. We identified points of resistance and inertia in the face of change at a local clinic level, and how lesser skilled staff cadres, such as counsellors, felt the brunt of change. A CAS analysis also revealed the patterns in the system that could inhibit change, such as negative feedback loops and path dependency, but also the positive effects of networks, individual nodal actors, and the power of self-organisation and emergent behaviour. The health system environment and characteristics had a clear bearing on the direction of change, such as through work cultures defined by reactive working rather than proactive planning. The value of a dedicated leadership in motivating for the benefits of the model to those involved further down the chain of decisions in order to achieve implementation, was an enabling factor in sustaining change [19, 23].

The political pressure for change within the health system pushed the scaling up of the innovation. Given that policy-makers are often working in an environment that exerts pressure to pursue change before conclusive evidence and outcomes are evident, the monitoring of the process of change is particularly pertinent, with a weighing up of risks and benefits of change [36]. In this regard, a clear agreement of the initial principles and goals of intervention and the framing of the problem is important to refer back to as trade-offs emerge in the process of scaling up. An argument for ensuring a wide range of outcome measures for assessing the ‘success’ of change is especially pertinent when change involves a complex organisational intervention. Metrics can obscure the multi-level dynamics of systems by focusing on sub-systems within the whole [21]. Retention-in-care and viral load measures, as well as the performance target of recruitment percentages, had come to be seen as the proxies for club functioning and for quality of care. The chosen indicators did not yet include process indicators for scaling up and broader systems objectives. In this regard, the way in which the nature of change is conceptualised becomes significant to consider. The idea of ‘innovation’ in policy as a discursive trope can increase the impression that change will inevitably have a positive effect, and can inadvertently stigmatise the reactions of those who express concerns about change. The interplay of a discourse of resilience and innovation might create an expectation that systems should embrace change through a combination of flexibility, ingenuity and technical brilliance. Sheikh et al. [22 pg 4] warn against the supremacy of a positivist paradigm in the evaluation of outcomes of change in health systems, which they argue “has led to health systems being seen primarily as vehicles for technological solutions rather than being grounded in political and social contexts with underlying power structures, interests, and interdependencies”. The framing of a complex organisational intervention as an ‘innovation’ in policy rhetoric can thus have profound implications for the assumptions about the effort and resources required to achieve change, and for the ways in which success is defined and measured. As our study made abundantly clear, the health system has sets of social practices and relationships and organisational cultures that have been concretised over time. This includes relations between staff, and also the relations of care with those using the system, rooted in local economies of care. These have to be taken seriously to ensure that staff accept the benefits of innovation and in order for change to be positive, acceptable and sustainable.

A limitation of our study was that we did not speak to people enrolled in the ART programme who were *not* in clubs, in order to also elicit their views on the privileges given to others, and the fairness of the process of scaling up. We would argue that a more fine-grained study is needed of equity outcomes and quality concerns of scaling up [32] in the case of the club model. New systems of care can introduce new inequalities despite the best intentions. Who are the kinds of patients who are considered “responsible” and meet the criteria for club entry? Who are those excluded? Should aspects such as convenience and shorter queues be assigned as a privilege, or should more effort be made to ensure that these dimensions of quality of care are possible, as a right for all people using the public sector? Differentiated care had been promoted to HIV positive people as more convenient care, possibly at the expense of stressing the actual underlying principle of ensuring the most appropriate level of attention for clinical status. The ethos of reward that lingered in the idea of the club members as “VIP patients” still created a sense of relegation from clubs as punishment, rather than an appropriate return to greater intensity of care from clinicians. Patients, such as pregnant women, thus resisted leaving clubs even to the detriment of their biomedical care. As the number of people in clubs increased dramatically with the scaling up and the lowering of criteria, the elitist aspect of club membership was diluted. We would argue that this constituted a positive unintended consequence of the scaling up of the club model.

## Conclusions

We have argued that innovation in largescale, complex programmes in health systems is a continuous process that requires ongoing support and ongoing attention to new innovation as challenges emerge. Our study suggests that it cannot be assumed that, beyond initial roll-out, further scaling up of a model will ‘take care of itself’. An innovation that appeared as an excellent idea and which was working very well whilst supported on a small scale, could readily be overwhelmed by the scale of change as margins for flexibility closed, vulnerabilities magnified and the capacity for adaption was exceeded. Rapid scaling up is also likely to require recourse to further resources, human or otherwise, and a culture of iterative learning to address emerging challenges and mitigate complex system errors. We would argue that ongoing support, and a systematic and formal process to identify and institutionalise the necessary innovation to consolidate and embed a largescale programme, would be a necessary step to the future success of ART clubs as a cornerstone of differentiated care. A culture of learning and monitoring of a range of process and outcome indicators can enable realistic approaches to the pace and scale of change and realistic assessments of the capacities of facility staff for change. At the same time, the study reveals the impressive capacity that a health system, and dedicated staff within such a system, can have for catalysing novel approaches. The study illustrated the drive for change directed from above but also a responsiveness to facility-level solutions. Significant learning has been garnered from the club experience that has relevance for the ongoing efforts to develop differentiated care for HIV and also for the urgent issue of providing the same level of support for people with other chronic lifelong conditions in low-resource settings. Further research is needed to assess the equity and quality outcomes of a differentiated care model and to ensure the inclusive distribution of the benefits to all categories of people living with HIV.

## Abbreviations

ART: Anti-retroviral Therapy
CAS: Complex Adaptive Systems
CCTDoH: City of Cape Town Department of Health
CDU: Chronic Dispensing Unit for pharmaceuticals, Western Cape
CHW: Community Health Worker
CI: Confidence Interval
HAST: HIV, AIDS, STIs, TB (service)
HIV: Human Immunodeficiency Virus
HMIS: Health Management Information System
IHI: Institute for Healthcare Improvement
LMIC: Low and middle-income country
LTFU: Lost To Follow Up
MSF: Médecins Sans Frontières
NCD: Non-communicable Disease
NGO: Non-governmental Organisation
QPUP: Quick-Pick-Up (of medication)
SOP: Standard Operating Procedure
VL: Viral Load measurement
WCDoH: Western Cape Provincial Department of Health
WHO: World Health Organisation

## Footnotes

1 One-year estimates of retention-in-care show a fall over 10 years from 85.1% to 77% in 2011 [3].

2 Both the province and city departments of health provide HIV and TB services in the Cape Town metropolitan area, with facilities allocated between them. Our study included facilities under both jurisdictions.

3 Other interviewees reported similar experiments, e.g. by the NGO TB/HIV care in the area of Gugulethu. A doctor who had worked in a high burden facility indicated that staff members had tried to institute their own system to streamline the service.

4 This included programme managers from the provincial and city health authorities, as well as key MSF and IHI staff.

5 People in clubs are allowed to authorise someone else to collect their medication if it is not a clinical or blood visit.

6 Staff cadres identified for involvement in clubs included doctors, nurses (with the club coordinator often a nurse), HIV counsellors, pharmacy assistants, and a data clerk.

7 This further streamlines proceedings and reduces duplication. In some facilities, the club folders are filed separately for easy access in case a nurse opinion and subsequent clinical note is required.

8 One example was a club where counsellors had started checking the identity documents of members before issuing their medication, which was time consuming and slowed down the club.

9 This has been instituted in the national central chronic medicine dispensing and distribution programme.

## References

[1] Boulle A, Schomaker M, May M, Hogg R, Shepherd B, Monge S, Keiser O, Lampe F, Giddy J, Ndirangu J, Garone D, Fox M, Ingle S, Reiss P, Dabis F, Costagliola D, Castagna A, Ehren K, Campbell C, Gill M, Saag M, Justice A, Guest J, Crane H, Egger M, Sterne J. Mortality in patients with HIV-1 infection starting antiretroviral therapy in South Africa, Europe, or North America: a collaborative analysis of prospective studies. PLoS Med. 2014;11(9). 10.1371/journal.pmed.1001718. 10.1371/journal.pmed.1001718 PMC4159124 25203931

[2] Boulle A (2008). Antiretroviral therapy and early mortality in South Africa. Bull World Health Organ.

[3] Osler M, Hilderbrand K, Hennessey C, Arendse J, Goemaere E, Ford N, Boulle A (2014). A three-tier framework for monitoring antiretroviral therapy in high HIV burden settings. J Int AIDS Soc.

[4] du Plessis J (2008). The chronic dispensing unit. SA Pharm J.

[5] Thompson M, Mugavero M, Amico K, Cargill V, Chang L, Gross R, Orrell C, Altice F (2012). Guidelines for improving entry into and retention in care and antiretroviral adherence for persons with HIV: evidence-based recommendations from an International Association of Physicians in AIDS care panel. Ann Intern Med.

[6] Nordling L (2016). South Africa ushers in a new era for HIV: the country has developed the biggest programme of antiretroviral therapy in the world. Now scientists are exploring the long-term consequences of the drugs. Nature.

[7] South African National Department of Health. Annual Report. 2015/16. Pretoria: National Department of Health.

[8] Meintjes G, Kerkhoff A, Burton R, Schultz C, Boulle A, Van Wyk G, Blumenthal L, Nicol M, Lawn S (2015). HIV-Related Medical Admissions to a South African District Hospital Remain Frequent Despite Effective Antiretroviral Therapy Scale-Up. Medicine.

[9] Boulle A, Van Cutsem G, Hilderbrand K, Cragg C, Abrahams M, Mathee S, Ford N, Knight L, Osler M, Myers J, Goemaere E, Coetzee D, Maartens G (2010). Seven-year experience of a primary care antiretroviral treatment programme in Khayelitsha, South Africa. AIDS.

[10] Coetzee D, Boulle A, Hildebrand K, Asselman V, Van Cutsem G, Goemaere E (2004). Promoting adherence to antiretroviral therapy. AIDS.

[11] Haberer JE, Sabin L, Amico KR, Orrell C, Galárraga O, Tsai AC, Vreeman RC, Wilson I, Sam-Agudu NA, Blaschke TF, Vrijens B, Mellins CA, Remien RH, Weiser SD, Lowenthal E, Stirratt MJ, Sow PS, Thomas B, Ford N, Mills E, Lester R, Nachega JB, Bwana BM, Ssewamala F, Mbuagbaw L, Munderi P, Geng E, Bangsberg DR (2017). Improving antiretroviral therapy adherence in resource-limited settings at scale: a discussion of interventions and recommendations. J Int AIDS Soc.

[12] Grimsrud A, Lesosky M, Kalombo C, Bekker LG, Myer L (2016). Community-based adherence clubs for the Management of Stable Antiretroviral Therapy Patients in cape town, South Africa: a cohort study. J Acquir Immune Defic Syndr.

[13] Grimsrud A, Sharp J, Kalombo C, Bekker LG, Myer L (2015). Implementation of community-based adherence clubs for stable antiretroviral therapy patients in cape town, South Africa. J Int AIDS Soc.

[14] Holmes CB, Sanne I (2015). Changing models of care to improve progression through the HIV treatment cascade in different populations. Curr Opin HIV AIDS.

[15] Adam T, de Savigny D (2012). Systems thinking for strengthening health systems in LMICs: need for a paradigm shift. Health Policy Plan.

[16] Peters D (2014). Advancing the application of systems thinking in health: why use systems thinking?. Health Res Policy Syst.

[17] Paina L, Peters D (2012). Understanding pathways for scaling up health services through the lens of complex adaptive systems. Health Policy Plan.

[18] de Savigny D, Adam T (2009). Systems Thinking for Health Systems Strengthening. Alliance for Health Policy and Systems Research.

[19] Swanson R, Cattaneo A, Bradley E, Chunharas S, Atun R, Abbas KM, Katsaliaki K, Mustafee N, Meier BM, Best A (2012). Rethinking health systems strengthening: key systems thinking tools and strategies for transformational change. Health Policy Plan.

[20] Adam T (2014). Editorial: advancing the application of systems thinking in health programmes. Health Res Policy Syst.

[21] Dattée B, Barlow J (2010). Complexity and whole-system change. J Health Serv Res Policy.

[22] Sheikh K, Gilson L, Agyepong IA, Hanson K, Ssengooba F (2011). Building the Field of Health Policy and Systems Research: Framing the Questions. PLoS Med.

[23] Gilson L, Barasa E, Nxumalo N, Cleary S, Goudge J, Molyneux S, Tsofa B, Lehmann U, Gilson L, et al. Everyday resilience in district health systems: emerging insights from the front lines in Kenya and South Africa BMJ Glob Health*.* 2017; doi: 10.1136/bmjgh-2016-000224. 10.1136/bmjgh-2016-000224 PMC5656138 29081995

[24] Gilson L, Hanson K, Sheikh K, Agyepong IA, Ssengooba F (2011). Building the Field of Health Policy and Systems Research: Social Science Matters. PLoS Med.

[25] MacGregor H, Bloom G (2016). Health systems research in a complex and rapidly changing context: ethical implications of major health systems change at scale. Dev World Bioeth.

[26] RESYST Research Consortium Key Findings Sheet. What is everyday health system resilience and how might it be nurtured? (n.d). 2016.

[27] Witter S and Hunter B. ‘Resilience of health systems during and after crises – what does it mean and how can it be enhanced?’ Health systems during and after crisis: evidence for better policy and practice - brief 1. Rebuild consortium (research for stronger health systems during and after crisis). 2017.

[28] Gilson L, Elloker S, Olckers P, Lehmann U (2014). Advancing the application of systems thinking in health: south African examples of a leadership of sense-making for primary health care. Health Res Policy Syst.

[29] Elloker S, Olckers P, Gilson L, Lehmann U. Crises, Routines and Innovations: The complexities and possibilities of sub-district management. South Afr Health Rev. 2013;275-173.

[30] Subramanian S, Naimoli J, Matsubayashi T, Peters DH (2011). Do we have the right models for scaling up health services to achieve the millennium development goals?. BMC Health Serv Res.

[31] Hanson K, Cleary S, Schneider H, Tantivess S, Gilson L (2010). Scaling up health policies and services in low- and middle-income settings. BMC Health Serv Res.

[32] Mangham LJ, Hanson K (2010). Scaling up in international health: what are the key issues?. Health Policy Plan.

[33] Peters D, Tran N, Adam T (2013). Implementation Research in Health: A Practical Guide.

[34] Atun R (2012). Health systems, systems thinking and innovation. Health Policy Plan.

[35] Greenhalgh T, Glenn R, MacFarlane F, Bate P, Kyriakidou O (2004). Diffusion of innovations in service organizations: systematic review and recommendations. Millbank Q.

[36] Denis JL, Hébert Y, Langley A, Lozeau D, Trottier LH (2002). Explaining diffusion patterns for complex health care innovations. Health Care Manag Rev.

[37] Leach M, Rockström J, Raskin P, Scoones I, Stirling AC, Smith A, Thompson J, Millstone E, Ely A, Arond E, Folke C, Olsson P (2012). Transforming innovation for sustainability. Ecol Soc.

[38] Smith A, Stirling A (2018). Innovation, sustainability and democracy: an analysis of grassroots contributions. J Self Governance Manage Econ.

[39] Douthwaite B, Ashby J (2005). Innovation histories: a method for learning from experience. The Institutional Learning and Change (ILAC) Initiative.

[40] MacGregor H, McKenzie A, Jacobs T, Ullauri A (2016). Analysis of Factors affecting the scale up and roll out of Adherence Clubs in the Cape Town Metro.

[41] Bemelmans M, Baert S, Goemaere E, Wilkinson L, Vandendyck M, Cutsem G, Kalenga L (2014). Community-supported models of care for people on HIV treatment in sub-Saharan Africa. Tropical Med Int Health.

[42] Decroo T, Telfer B, Biot M, Maikere J, Dezembro S, Cumba L (2011). Distribution of antiretroviral treatment through self-forming groups of patients in Tete Province, Mozambique. J Acquir Immune Defic Syndr.

[43] Pellecchia U, Baert S, Nundwe S, Bwanali A, Zamadenga B, Metcalf CA, Bygrave H, Daho S, Ohler L, Chibwandira B, Kanyimbo K (2017). We are part of a family. Benefits and limitations of community ART groups (CAGs) in Thyolo, Malawi: a qualitative study. J Int AIDS Soc.

[44] Institute of Healthcare Improvement (2003). Innovation series 2003 (the breakthrough series): IHI’s collaborative model for achieving breakthrough improvement.

[45] Wilkinson L (2013). ART adherence clubs: A long-term retention strategy for clinically stable patients receiving antiretroviral therapy. South Afr J HIV Med.

[46] Luque-Fernandez MA, Van Cutsem G, Goemaere E, Hilderbrand K, Schomaker M, Mantangana N (2013). Effectiveness of patient adherence groups as a model of care for stable patients on antiretroviral therapy in Khayelitsha, Cape Town. South Africa. PLoS One..

[47] Bango F, Ashmore J, Wilkinson L, Cutsem G, Cleary S (2016). Adherence clubs for long-term provision of anti-retroviral therapy: cost-effectiveness and access analysis from Khayelitsha, South Africa. Tropical Med Int Health.

[48] Tsondai P, Wilkinson L, Grimsrud A, Mdlalo P, Ullauri A, Boulle A (2017). High rates of retention and viral suppression in the scale-up of antiretroviral therapy adherence clubs in cape town, South Africa. J Int AIDS Soc.

[49] South African National Department of Health (2016). Adherence guidelines for HIV, TB, and NCDs: policy and service delivery guidelines for linkage to care, adherence to treatment and retention in care.

[50] Van Damme W, Kober K, Kegels G (2008). Scaling up antiretroviral treatment in southern African countries with human resource shortage: how will health systems adapt?. Soc Sci Med.

